# Association between free thyroxine and aggression: the chain mediating role of hostile attribution bias and psychological resilience

**DOI:** 10.3389/fpsyg.2026.1822609

**Published:** 2026-07-30

**Authors:** Yalan Wang, Ting Wang, Weixia Yu, Jianzheng Cai, Yingying Zhang, Yajie Ying

**Affiliations:** 1 Department of Nursing, The First Affiliated Hospital of Soochow University, Suzhou, China; 2 Department of Intensive Care Medicine, The First Affiliated Hospital of Soochow University, Suzhou, China; 3 Department of Chemotherapy, The First Affiliated Hospital of Soochow University, Suzhou, China

**Keywords:** aggression, free thyroxine, hostile attribution bias, psychological resilience, thyroid diseases

## Abstract

**Objective:**

Serum free thyroxine (FT_4_) has been demonstrated to be associated with human aggression. Based on the theoretical and empirical research on hostile attribution bias and psychological resilience, this study developed a chain mediating model to investigate whether these factors mediate the relationship between serum FT_4_ and aggression among thyroid disease patients.

**Methods:**

A total of 418 thyroid disease patients who met the inclusion and exclusion criteria were recruited from a general hospital in Suzhou, Jiangsu Province, China, to participate in this study and completed the self-report versions of the Buss-Perry Aggression Questionnaire (BPAQ), the Word Sentence Association Paradigm for Hostility Scale (WSAP-Hostility), and the Connor-Davidson Resilience Scale (CD-RISC10). Blood samples were collected to measure serum FT_4_ levels by chemiluminescence immunoassay. SPSS 32.0 was used for descriptive and inferential statistical analyses. Amos 29.0 was applied to conduct structural equation modeling to examine the chain mediating roles of hostile attribution bias and psychological resilience in the association between serum FT_4_ and aggression.

**Results:**

Serum FT_4_ levels were directly associated with aggression among thyroid disease patients (*β* = 0.244, 95% CI [0.148, 0.334]). Serum FT_4_ exhibited indirect statistical associations with aggression through two independent mediating pathways of hostile attribution bias (*β* = 0.129, 95% CI [0.088, 0.178]) and psychological resilience (*β* = 0.068, 95% CI [0.038, 0.107]), as well as the chain mediating pathway of these two variables (*β* = 0.029, 95% CI [0.016, 0.048]). Multi-group structural equation modeling demonstrated measurement and structural invariance across sexes, with no significant differences in the path coefficients.

**Conclusion:**

Hostile attribution bias and psychological resilience served as mediators in the association between serum FT_4_ levels and aggression among thyroid disease patients. Causal inferences cannot be drawn from this cross-sectional design, and future longitudinal studies are required to extend these findings.

## 1 Introduction

As one of the most important indices for assessing thyroid function, serum free thyroxine (FT_4_) is produced by the thyroid gland and plays a crucial role in various physiological processes, including metabolism and brain development, across multiple organs and systems (Chaker et al., 2018). Maintaining optimal FT_4_ levels is essential for the normal functioning of these systems, particularly during nervous system development and in the regulation of mood and behavior, including aggression (Kuś et al., 2021). FT_4_, in relation to aggression, has been studied mostly in patients with borderline personality disorder (Flach et al., 2021), whereas limited research has been conducted in thyroid disease patients.

Thyroid diseases caused by varying levels of FT_4_ are common and lead to thyroid dysfunction. These conditions mainly include hypothyroidism, characterized by decreased FT_4_ levels; hyperthyroidism, often due to Graves’ disease and marked by elevated FT_4_ levels; iodine deficiency disorders; thyroiditis; thyroid nodules; benign thyroid tumors; and thyroid cancer. Unlike endocrine glands such as the mammary glands, prostate, ovaries, and testes, the thyroid gland plays essential physiological roles in both males and females. As one of the most prevalent endocrine disorders, thyroid diseases affect approximately 3–21% of the global population (Canaris et al., 2000; Ochs et al., 2008; Tasnim and Nyholt, 2023; Zou et al., 2021). A previous epidemiological survey conducted in 31 cities in China reported that the overall prevalence of thyroid diseases was 50.96% (Liu and Shan, 2021). Considering the adverse consequences of aggression, the potential role of FT_4_ in human aggression, and fluctuations in FT_4_ levels among patients with thyroid diseases, it is important to explore the influencing factors and underlying mechanisms of aggression in this population.

According to the General Aggression Model (GAM) (Allen et al., 2018), the interaction of individual and situational factors activates the individual’s internal state, encompassing cognition, emotion, and their interplay, which in turn triggers behavioral outcomes. Drawing upon this framework, the present study proposes that serum FT_4_, as an important biological factor, may influence aggression by altering key components of the internal state. Hostile attribution bias, as an important cognitive factor (Dodge et al., 2015), and psychological resilience, as an important psychological factor (Qiao et al., 2024), have been demonstrated to be correlated with aggression in both theoretical and empirical studies. On this basis, the present study developed a chain mediating model to investigate whether hostile attribution bias and psychological resilience mediate the association between serum FT_4_ levels and aggression among thyroid disease patients.

### 1.1 FT_4_ and aggression

Research indicates that FT_4_ levels are positively correlated with scores on the Modified Overt Aggression Scale among males (Ma et al., 2022). Another study (Sinai et al., 2014) reported that women who experienced higher levels of childhood violence had lower FT_3_/FT_4_ ratios. Moreover, a positive correlation is observed between serum FT_4_ levels and aggression scores among patients with noncriminal antisocial personality disorder (APD), whereby elevated FT_4_ is accompanied by higher aggression (Evrensel et al., 2016). Additionally, changes in FT_4_ levels have been confirmed to be significantly positively correlated with alterations in aggression, including anger, violent ideation, and irritability (Daly et al., 2003). The potential mechanisms underlying the relationship between FT_4_ and aggression could involve excess thyroid hormone affecting neurotransmitters (serotonin, gamma-aminobutyric acid and dopamine) or second messengers (adenyl cyclase and phospholipase-C systems). According to a systematic review conducted in Switzerland, higher FT_4_ levels are associated with lower anxiety levels in patients with panic disorder, which may indirectly influence their aggression (Fischer and Ehlert, 2018). Taken together, existing findings suggest that serum FT_4_ levels may be associated with aggression. However, previous studies investigating the relationship between FT_4_ levels and violent behaviors have mainly focused on male schizophrenics (Yu et al., 2024), male normal volunteers (Daly et al., 2003), or patients with noncriminal APD (Evrensel et al., 2016), rather than thyroid disease patients who experience substantial fluctuations in thyroid hormone levels. Therefore, the association between FT_4_ levels and human aggression requires further investigation. This study proposes the hypothesis 1: Serum FT_4_ levels are positively associated with aggression among thyroid disease patients.

### 1.2 The mediating role of hostile attribution bias

As an indicator for measuring cognitive processing methods, hostile attribution bias (HAB) is a significant indicator for evaluating individual social information processing (Dodge, 2006). It raises great public concern and has become an important predictor of aggression (Tuente et al., 2019). Specifically, individuals with higher levels of hostile attribution bias tend to interpret ambiguous social information as threatening or hostile, thereby eliciting negative emotional responses, such as anger, and subsequently increasing the likelihood of aggressive behaviors. For example, previous research has shown that when individuals tend to interpret others’ ambiguous behaviors as hostile in social situations, this cognitive bias initiates higher aggressive responses (Liu et al., 2024). This finding aligns with earlier research conclusions that hostile attribution bias is primarily associated with reactive aggression (de Castro et al., 2005).

Research has found that interventions targeting hostile attribution bias can reduce aggressive behavior to some extent, although their effects are often limited (Hudley and Graham, 1993). A longitudinal study conducted by Godleski and Ostrov (2010) revealed that hostile attribution bias among third- to fifth-grade primary school students could predict their aggression in the sixth grade. Dodge et al. (2015) carried out a cross-cultural study involving 1,299 children from 12 different countries. The results of longitudinal mediating effect analysis indicate that after controlling for children’s early aggression, hostile attribution bias acting as a mediating variable longitudinally predicted aggression 1 year later. In addition, a randomized controlled trial involving 80 patients demonstrates that hostile attribution bias was positively associated with human aggression (Liu et al., 2024). A previous cross-sectional study (Barbero et al., 2015) involving young patients with early psychosis (aged 18–35 years; illness duration <3 years) reports that cognitive performance in the domains of attention and social cognition, including hostile attribution bias as a key component, is statistically associated with FT_4_ levels but not with TSH levels or thyroid antibodies. Therefore, we hypothesize that hostile attribution bias may play a mediating role between FT_4_ and aggression in thyroid disease patients. Based on the above analysis, this study proposes the hypothesis 2: Hostile attribution bias is positively associated with aggression and plays a mediating role in the association between serum FT_4_ levels and aggression among thyroid disease patients.

### 1.3 The mediating role of psychological resilience

In addition to this cognitive pathway, the present study also proposes that psychological resilience, a positive psychological resource, may serve as another mediator in this association. As a positive psychological resource, psychological resilience refers to the ability to cope with adversity, including stress, difficulties, and setbacks (Tay and Lim, 2020). It has been shown that individuals with higher levels of psychological resilience have greater emotional regulation abilities and are better able to maintain psychological balance, which may contribute to lower levels of anxiety, depression, and even aggression (Imran et al., 2024). For decades, thyroid hormones have been investigated in relation to depression and anxiety disorders (Fischer and Ehlert, 2018; Bauer and Whybrow, 2021), which could indicate a certain impact on psychological resilience.

However, few studies have examined the association between FT_4_ levels and psychological resilience among thyroid disease patients. A study conducted among Chinese college students indicates that low psychological resilience, which refers to a lack of effective coping abilities and adaptability to respond to adversity, is positively related to aggression (Li et al., 2022). Similarly, psychological resilience levels are associated with aggression among young people (including college students, community youths, migrant workers, and so on) in China (*r* = −0.18, *p* < 0.05) (Zhang et al., 2021). Based on these findings, this study proposes the hypothesis 3: Psychological resilience is negatively associated with aggression and plays a mediating role in the association between serum FT_4_ levels and aggression among thyroid disease patients.

### 1.4 The relationship between hostile attribution bias and psychological resilience

Given the complex interplay between cognitive and psychological factors, the present study proposes a chain mediating model in which hostile attribution bias and psychological resilience play a serial mediating role in the relationship between serum FT_4_ levels and aggression in thyroid disease patients.

The Social Information Processing (SIP) Model (Crick and Dodge, 1994) specifies a six-step cognitive sequence through which individuals interpret and respond to social situations: (a) encoding of cues, (b) interpretation of cues, (c) goal clarification, (d) response search, (e) response decision, and (f) behavioral enactment. Critically, this is a temporally ordered sequence in which earlier steps provide the foundation for later steps. According to the SIP Model, hostile attribution bias occurs at Step b (interpretation of social cues)—the earliest cognitive gateway in the processing stream, where individuals assign meaning to social information before any behavioral response has been generated. This interpretation forms the basis upon which all subsequent steps operate. Psychological resilience, in contrast, functions as a higher-order regulatory resource that primarily operates at later stages (Steps c and d: response search and decision), where it facilitates the selection of non-aggressive coping strategies. Thus, within the SIP Model, the temporal positions of the two constructs are clearly differentiated. Consequently, because hostile interpretation (Step b) must occur before response generation and selection (Steps d and e), the SIP Model’s sequential logic dictates that hostile attribution bias temporally precedes psychological resilience. Furthermore, sustained hostile interpretation consumes cognitive and emotional resources, thereby depleting the regulatory capacity that sustains resilience—a notion consistent with the ego depletion theory in emotion regulation literature (Baumeister et al., 2024).

This theoretical sequencing is further supported by empirical evidence. Studies have shown that psychological resilience is significantly negatively correlated with hostile attribution bias. In other words, individuals with higher levels of psychological resilience are better able to cope with frustration in a more positive and calm manner, rather than responding through hostile attribution (Wang et al., 2024). On the contrary, lower levels of psychological resilience result in poorer adaptability and a greater susceptibility to being affected by negative emotions, which increases the tendency toward hostile attribution bias (Labrague, 2021). Moreover, psychological resilience has been shown to be associated with depressive symptoms, and may also predispose individuals to engage in riskier behaviors when facing stressful environments, thereby further contributing to the occurrence of aggressive behavior (Li et al., 2022). In summary, hostile attribution bias serves as the early maladaptive cognitive response, while psychological resilience functions as a subsequent adaptive resource to counteract this bias. Based on these findings, this study proposes the hypothesis 4: Hostile attribution bias is negatively associated with psychological resilience, and hostile attribution bias and psychological resilience play a serial mediating role in the association between serum FT_4_ levels and aggression among thyroid disease patients.

Taken together, a substantial body of empirical studies suggests that serum FT_4_ levels are associated with aggression; however, the underlying mechanism of this association remains unclear. The present study aimed to extend previous research by investigating the association between serum FT_4_ levels and aggression among thyroid disease patients and testing the mediating roles of hostile attribution bias and psychological resilience. This study will not only enrich and expand the research on serum FT_4_ levels and aggression but also hold significant theoretical value and practical significance for understanding and intervening in aggression in this population.

## 2 Methods

### 2.1 Study design and setting

This study employed a cross-sectional correlational design. The data were collected through convenience sampling at a general hospital in Suzhou, Jiangsu Province, China from January 2023 to July 2024. Hospitalized thyroid disease patients who had complied with the inclusion and exclusion criteria were recruited.

The recruitment of hospitalized patients offered two key methodological advantages. First, this sample guaranteed high diagnostic consistency. All participants received standardized in-hospital tests to ensure accurate thyroid disease diagnosis, effectively eliminating diagnostic ambiguity, self-report bias, and unrecorded medication confounding prevalent in community samples dependent on self-reported medical histories. Second, the inpatient setting provided a standardized environment for assessing the core outcome of aggression. The ward offered consistent situational stimuli and fixed behavioral targets (i.e., healthcare workers), thereby minimizing environmental confounders that are difficult to control in diverse community settings. In this study, aggression was measured using the Buss-Perry Aggression Questionnaire (BPAQ; Buss and Perry, 1992). Its four subdomains—physical aggression, verbal aggression, anger, and hostility—assess trait aggression, which was considered a dispositional precursor to aggressive behavior toward healthcare workers in hospital settings. The BPAQ was therefore a suitable measure for this sample.

### 2.2 Inclusion and exclusion criteria

Participants were included if they met the following criteria: (1) they had a diagnosis according to the Clinical Guidelines for the Management of Thyroid Disorders in China (see the attachment for details); (2) their Mini Mental State Examination (Folstein et al., 1975) scores met the education-adjusted cutoffs: >17 for illiterate patients, >20 for patients with a primary school education, and >24 for those with junior high school education or higher; (3) they were able to understand the questionnaires and communicate verbally; and (4) they provided written informed consent and voluntarily agreed to participate. Exclusion criteria were as follows: (1) presence of other endocrine system diseases (affecting the adrenal and gonadal axes), severe cardiovascular and cerebrovascular diseases, or psychiatric disorders (including depression, anxiety, and other mental illnesses); (2) consciousness disorders, severe auditory or visual impairments, or aphasia; (3) current use of antidepressants, antipsychotics, or anxiolytics, or medications affecting thyroid hormone levels (e.g., lithium salts); (4) pregnancy or lactation; and (5) questionnaires with missing data, incomplete responses, contradictory answers, or inaccurate entries.

### 2.3 Sample size estimation

According to the sample size estimation method for structural equation modeling, the recommended minimum sample size is 200 cases (Kline, 2016). Considering a 10% non-response rate, we determined a minimum target of 223 participants. The final sample comprised 418 participants, meeting this requirement.

### 2.4 Measures

After blood collection for thyroid hormone assays, which included FT_4_, thyroid-stimulating hormone (TSH), triiodothyronine (T_3_) and free triiodothyronine (FT_3_), all participants completed structured questionnaires, a process that took approximately 20 min. The questionnaires collected demographic information and comprised three validated scales: the Buss-Perry Aggression Questionnaire (BPAQ) (Buss and Perry, 1992), the Word Sentence Association Paradigm for Hostility Scale (WSAP-Hostility) (Dillon et al., 2016), and the Connor-Davidson Resilience Scale (CD-RISC-10) (Wang et al., 2010).

#### 2.4.1 Variables and instruments

Sociodemographic information of the participants was collected using a standardized questionnaire, including sex, marital status, whether the participant was an only child, educational level, disease duration, medical payment method, perceived financial burden of medical costs, healthcare satisfaction, and level of trust in medical workers.

#### 2.4.2 Serum FT_4_, TSH, T_3_ and FT_3_

Within the first 24 h of admission, fasting morning blood samples (between 6:00 and 8:00 a.m.) were obtained to determine thyroid hormone concentrations, in order to control for the influence of circadian secretory rhythms on the results. Five milliliters of fasting elbow venous blood was collected into an anticoagulant tube and centrifuged to obtain serum (3,000 rpm, 3–5 min). Serum FT_4_, TSH, T_3_ and FT_3_ levels were measured using a chemiluminescence immunoassay (Cobas e 601 immunoassay analyzer, Roche Diagnostics, Switzerland). All procedures were performed strictly in accordance with the manufacturer’s instructions (Roche). All blood samples were assayed in triplicate, and the mean value was used for analysis to minimize measurement error. The intra- and inter-assay coefficients of variability (CV) for results were both below 5%. The manufacturer-provided reference range for serum FT_4_ on the Cobas e 601 platform was 12.0–22.0 pmol/L, TSH was 0.27–4.2 mIU/L, T_3_ was 0.8–2 ng/mL and FT_3_ was 3.1–6.8 pmol/L.

#### 2.4.3 Buss-Perry aggression questionnaire (BPAQ)

The BPAQ (Buss and Perry, 1992) is a psychological measurement tool developed by psychologists Arnold H. Buss and Mark Perry in 1992 to assess individual trait aggression through self-report. It has been widely used in psychological research and clinical assessments. Its reliability and validity in the Chinese context have been confirmed by Gao et al. (2023). The scale comprises four dimensions: physical aggression, verbal aggression, anger and hostility. It consists of 29 items, each rated on a 5-point Likert scale (1 = very inconsistent, 2 = relatively inconsistent, 3 = unclear, 4 = fairly consistent, 5 = very consistent). This instrument was used to measure participants’ aggression. Higher total scores indicate greater levels of aggression. In this study, the Cronbach’s *α* for the BPAQ was 0.879.

#### 2.4.4 Word sentence association paradigm for hostility scale (WSAP-hostility)

The WSAP-Hostility (Dillon et al., 2016) is a well-validated and reliable instrument commonly used to assess hostile attribution bias in specific situations. The scale consists of 32 self-reported items, each comprising an ambiguous sentence paired with either a hostile or benevolent word. Participants were presented with each sentence–word pair and asked to rate the degree of relevance on a 6-point scale (1 = completely irrelevant, 2 = mostly irrelevant, 3 = slightly irrelevant, 4 = slightly relevant, 5 = mostly relevant, 6 = extremely relevant). The benevolent attribution subscale includes items 1, 4, 5, 7, 9, 10, 14, 17, 18, 20, 23, 25, 27, 29, and 30, whereas the hostile attribution subscale comprises items 2, 3, 6, 8, 11, 12, 13, 15, 16, 19, 21, 22, 24, 26, 28, 31, and 32. The difference between the hostile and benevolent subscale scores reflects the level of hostile attribution bias, with higher difference scores indicating a greater level of hostile attribution bias. In this study, the Kaiser–Meyer–Olkin measure was 0.669, and Bartlett’s test of sphericity was significant (*χ*^2^ = 7066.578, *df* = 496, *p* < 0.001). Nine factors with eigenvalues >1 were extracted, accounting for 69.48% of the total variance. All items had communalities >0.5, and most items showed primary factor loadings >0.5 after rotation, indicating acceptable structural validity. The Cronbach’s *α* coefficients for the WSAP-Hostility, the hostile attribution subscale and the benevolent attribution subscale were 0.743, 0.806 and 0.793, respectively.

#### 2.4.5 Connor-Davidson resilience scale (CD-RISC-10)

The CD-RISC*-*10, originally developed by Connor and Davidson (2003) and later translated and adapted for the Chinese population by Wang et al. (2010), was used to assess participants’ psychological resilience. The scale comprises 10 items, each rated on a 5-point Likert scale ranging from 1 (never) to 5 (almost always). Total scores range from 10 to 50, with higher scores indicating greater psychological resilience. The Cronbach’s *α* for the CD-RISC-10 was 0.900 in this study.

### 2.5 Ethical considerations

This study was conducted in accordance with the Declaration of Helsinki and was approved by the Hospital Ethics Committee (No. 2022253). The researchers provided participants (*N* = 418) with ample opportunities to ask questions and receive clarifications throughout the study. An informed consent confirmation form was provided to ensure that participants fully understood its content before signing. Participants had the right to withdraw at any time without any consequences for their medical care.

### 2.6 Data analysis

The data analysis was performed using IBM SPSS Statistics for Windows, version 32.0 (IBM Corp., Armonk, NY, United States) and Amos statistical software version 29.0 (IBM Corp.). Descriptive statistics were calculated for all variables: the mean and standard deviation (M ± SD) for numerical variables and frequency or percentage for categorical variables. *T*-tests and one-way analysis of variance (ANOVA) were performed to compare the differences among participants in each group. Pearson correlation coefficients were calculated to examine the bivariate relationships among serum FT_4_, TSH, T_3_ and FT_3_, hostile attribution bias, psychological resilience and aggression. Serum FT_4_, TSH, T_3_ and FT_3_ were treated as continuous variables in the main analyses. To provide clinical information, participants were stratified by the key variables to analyze aggression scores across different subgroups. Specifically, thyroid hormones were classified into below normal, normal, and above normal categories based on reference intervals (FT_4_: 12.0–22.0 pmol/L; TSH: 0.27–4.2 mIU/L; T_3_: 0.8–2 ng/mL; FT_3_: 3.1–6.8 pmol/L), while hostile attribution bias and psychological resilience were trichotomized into low, medium, and high groups according to score tertiles (medium cutoffs: −20–33 and 25–40, respectively).

SEM was conducted using Amos version 29.0 to test whether serum FT_4_ was associated with aggression and whether this relationship was mediated by hostile attribution bias and psychological resilience. Model fit was evaluated based on criteria: the ratio of chi-square to degrees of freedom (*χ*^2^/*df*) was below 3; the root mean square error of approximation (RMSEA) was below 0.08; and the normed fit index (NFI), relative fit index (RFI), incremental fit index (IFI), and comparative fit index (CFI) were all greater than 0.9. A bias-corrected bootstrapping procedure was adopted to test the indirect effects. An indirect effect was considered statistically significant if the 95% confidence interval (CI) did not contain zero (O'Brien and Yi, 2016). Statistical significance was set as *p* < 0.05 (two-tailed).

In addition, two alternative model specifications were tested: (1) reversing the order of the two mediators (i.e., psychological resilience—hostile attribution bias); and (2) a reverse causal model in which aggression served as the predictor and FT_4_ as the outcome. All three models—the hypothesized model and the two alternatives—were estimated using the same SEM procedures and evaluated with the same fit indices.

To evaluate the robustness of the hypothesized model against potential confounding by other thyroid indicators, three additional covariates—TSH, T_3,_ and FT_3_—were each individually entered into the original serial mediation model. In each adjusted model, the respective thyroid indicator was specified as a covariate predicting both mediators and the outcome, with its covariance with FT_4_ freely estimated. Model robustness was assessed by examining whether the original path coefficients remained significant and comparable in magnitude, and whether model fit indices remained within acceptable ranges.

To examine sex differences, sex-stratified multi-group SEM analysis was additionally performed. A hierarchical invariance test was performed by sequentially constraining measurement and structural weights, with model comparisons based on |ΔCFI| ≤ 0.01 and ΔRMSEA ≤ 0.015 (Cheung and Rensvold, 2002), alongside Chi-square difference tests (Byrne, 2010). Upon establishing invariance, sex-specific path coefficients with 95% bias-corrected bootstrap CIs (5,000 resamples) were reported. Additionally, a post-hoc power analysis based on sample distribution (44% male, 56% female; *α* = 0.05) was conducted to evaluate statistical sensitivity (Cohen, 1988).

### 2.7 Quality control

The standardized questionnaire was developed following consultation with experts on the research team. All investigators possessed sufficient professional knowledge to explain and clarify each question using standardized language. The BPAQ includes two reverse-scored items, which were designed to prevent participants from responding carelessly or arbitrarily, thereby improving the quality of the responses. During data collection, the completeness and quality of each questionnaire were carefully reviewed. Questionnaires were considered invalid and excluded if they met any of the following criteria: (1) an item was left blank; (2) more than one option was selected for a single item; (3) all items were selected with the same response option; or (4) more than three items contained missing values. In addition, logical inconsistencies and missing entries were identified using SPSS 32.0, and necessary corrections were made for incomplete responses where appropriate.

## 3 Results

### 3.1 Common method bias test and multicollinearity test

The Harman single-factor test (Podsakoff et al., 2003) was used to test for common method bias. The unrotated factor analysis revealed a total of 20 factors with eigenvalues greater than 1, and the first factor explained only 18.19% of the variance, which was less than the 40% threshold, suggesting that common method bias was not a significant concern in this data. The multicollinearity among predictor variables was evaluated by examining the variance inflation factor (VIF). The results showed that the tolerance values for all predictor variables ranged from 0.761 to 0.868 (≤0.1 indicates the presence of multicollinearity), and the VIF values ranged from 1.260 to 1.315 (≥10 indicates the presence of multicollinearity), confirming that multicollinearity was not an issue among the predictor variables.

### 3.2 Descriptive statistics and correlation analysis

Table 1 presented the means and standard deviations of the study population’s demographics, along with comparisons of aggression scores among participants with different sociodemographic characteristics. Supplementary stratified comparisons of aggression scores across categorical groupings of FT_4_, TSH, T_3_, FT_3_, hostile attribution bias, and psychological resilience were presented in Supplementary Table S1. Given the small sample size of the “Completely Distrustful” subgroup (*n* = 5) in the trust-related categorical comparisons, a sensitivity analysis was conducted by excluding these five cases, without changing the results (overall mean: 68.79 vs. 68.22; F: 84.21 vs. 90.72; Supplementary Table S2).

**Table 1:** Comparison of aggression scores among participants with different socio-demographic characteristics (*N* = 418).

Characteristics	Number of patients (%)	BPAQ scores	*t/F*	*p*
		M ± SD		
Sex			3.756^a^	<0.001
Male	183 (43.78)	72.53 ± 19.15		
Female	235 (56.22)	65.88 ± 16.33		
Only child			6.507^a^	<0.001
Yes	126 (30.14)	77.06 ± 18.95		
No	292 (69.86)	65.22 ± 16.21		
Marital status			1.611^b^	0.201
Single	50 (11.96)	72.98 ± 20.63		
Married	355 (84.93)	68.16 ± 17.61		
Divorced or others	13 (3.11)	69.77 ± 12.85		
Educational level			1.141^b^	0.320
Primary	192 (45.93)	69.94 ± 17.98		
Secondary	144 (34.45)	67.00 ± 16.71		
Tertiary	82 (19.62)	69.24 ± 19.64		
Disease course (months)			1.912^b^	0.127
<6	197 (47.14)	67.60 ± 15.38		
6–12	85 (20.33)	72.16 ± 21.96		
12–36	46 (11)	71.24 ± 14.03		
>36	90 (21.53)	66.96 ± 20.15		
Medical payment methods			0.290^a^	0.772
Medical insurance	377 (90.19)	68.71 ± 17.77		
Out-of-pocket	41 (9.81)	69.56 ± 19.31		
The impact of medical costs			56.082^b^	<0.001
Significant	47 (11.24)	94.81 ± 15.51		
Considerable	81 (19.38)	73.17 ± 15.44		
Moderate	124 (29.67)	65.16 ± 15.10		
Minor	108 (25.84)	58.23 ± 12.50		
Negligible	58 (13.87)	69.00 ± 14.33		
Healthcare satisfaction			34.690^b^	<0.001
Very dissatisfied	9 (2.15)	105.78 ± 19.92		
Somewhat dissatisfied	6 (1.44)	88.50 ± 15.96		
Neutral	52 (12.44)	73.85 ± 12.17		
Somewhat satisfied	175 (41.87)	73.57 ± 17.41		
Very satisfied	176 (42.10)	59.98 ± 14.20		
Level of trust in medical workers			84.211^b^	<0.001
Completely distrustful	5 (1.20)	115.60 ± 3.36		
Somewhat distrustful	19 (4.55)	100.79 ± 11.56		
Neutral	39 (9.33)	86.77 ± 13.99		
Somewhat trustful	160 (38.27)	70.39 ± 14.57		
Completely trustful	195 (46.65)	59.56 ± 12.43		

M, mean; SD, standard deviation; a, independent samples *t*-Test; b, analysis of variance.

Table 2 presented the means and standard deviations of serum FT_4_, TSH, T_3_, FT_3_, hostile attribution bias, psychological resilience and aggression, as well as the Pearson correlation coefficients among all study variables. Analyses revealed significant relationships among serum FT_4_, hostile attribution bias, psychological resilience and aggression (*p* < 0.001). Serum FT_4_ and hostile attribution bias were positively correlated with aggression. Psychological resilience was negatively correlated with aggression and serum FT_4_. Hostile attribution bias was positively associated with serum FT_4_ and negatively associated with psychological resilience. TSH was negatively correlated with aggression, whereas T_3_ and FT_3_ showed no significant correlations with aggression.

**Table 2:** Statistical description and linear correlations among the study variables.

Variables	M ± SD	1	2	3	4	5	6	7
1. Serum FT_4_ (pmol/L)	17.13 ± 6.14	1						
2. Serum TSH (mIU/L)	2.09 ± 1.41	−0.035	1					
3. Serum T_3_ (ng/mL)	1.06 ± 0.37	0.118^*^	0.179^***^	1				
4. Serum FT_3_ (pmol/L)	4.08 ± 1.76	0.336^***^	0.105^*^	0.184^***^	1			
5. Hostile attribution bias	−7.18 ± 19.78	0.272^***^	−0.006	0.051	0.054	1		
6. Psychological resilience	34.74 ± 6.77	−0.336^***^	0.060	−0.011	−0.042	−0.434^***^	1	
7. Aggression	68.79 ± 17.90	0.418^***^	−0.146^**^	−0.013	0.072	0.608^***^	−0.512^***^	1

FT_4_, free thyroxine; TSH, thyroid-stimulating hormone; T_3_, triiodothyronine; FT_3_, free triiodothyronine; M, mean; SD, standard deviation; ^*^p < 0.05; ^**^p < 0.01; ^***^p < 0.001.

### 3.3 Hostile attribution bias and psychological resilience as mediators of the relationship between FT_4_ and aggression

#### 3.3.1 Model fit and mediation analysis

The correlation analysis results met the necessary statistical criteria for further testing the mediating effects of hostile attribution bias and psychological resilience (Wen and Ye, 2014). The model fit was tested using Amos 29.0 and indices were presented in Table 3. The specific results were as follows: *χ*^2^/*df* = 2.827, RMSEA = 0.047, NFI = 0.951, IFI = 0.968, TLI = 0.937, CFI = 0.967. All fit indices of the initial model fell within the acceptable range.

**Table 3:** Model fit and its evaluation indicators.

Index	*χ^2^/df*	RMSEA	NFI	IFI	TLI	CFI
Evaluation indicators	<3.00	<0.08	>0.90	>0.90	>0.90	>0.90
Initial model	2.827	0.047	0.951	0.968	0.937	0.967
Alternative model 1	2.827	0.047	0.951	0.968	0.937	0.967
Alternative model 2	2.827	0.047	0.951	0.968	0.937	0.967

χ^2^, Chi-square; df, Degrees of freedom; RMSEA, Root Mean-Square Error of Approximation; NFI, Normed Fit Index; IFI, Incremental Fit Index; TLI, Tucker-Lewis Index; CFI, Comparative Fit Index.

**Figure 1:**
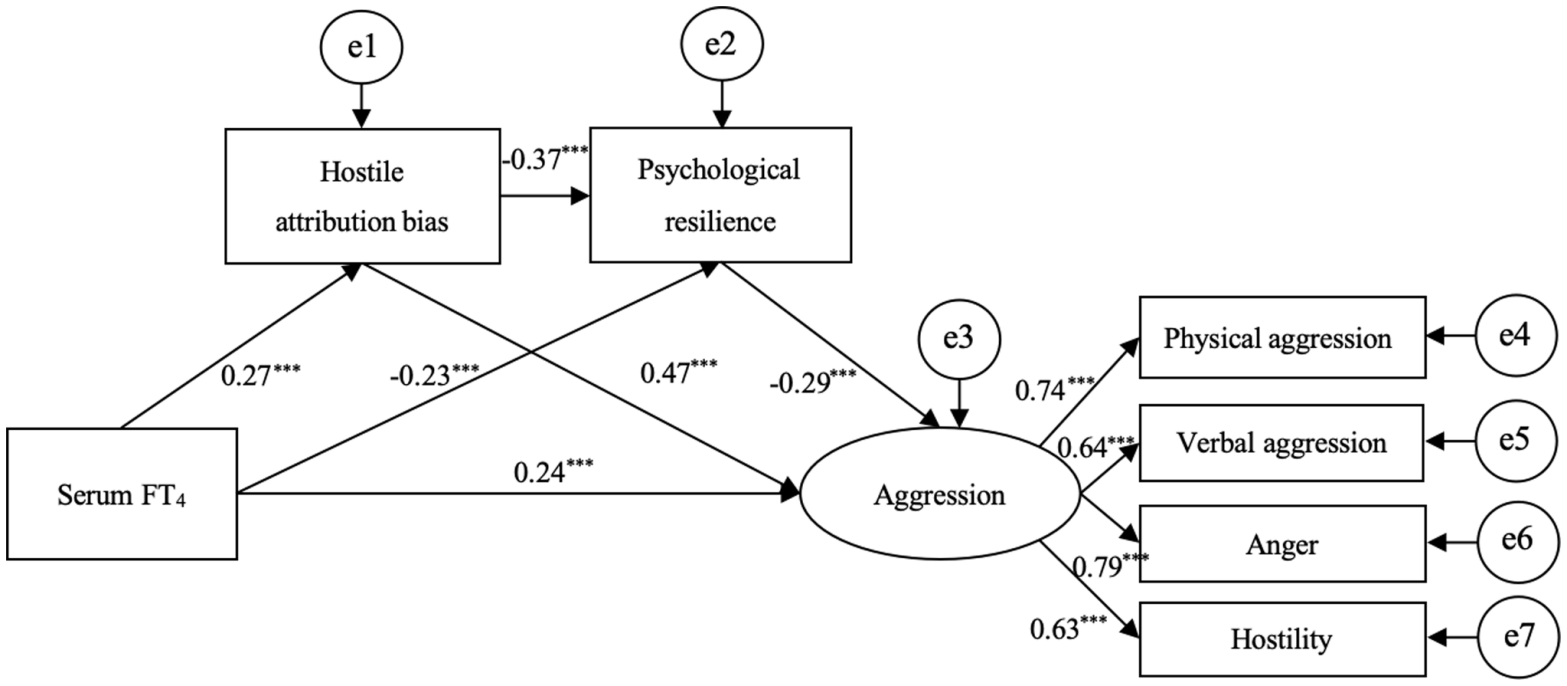
The final model of chain mediation model. ***p < 0.001.

The chain mediating pathway of hostile attribution bias and psychological resilience between serum FT_4_ and aggression in thyroid disease patients was presented in Figure 1. The results indicated that serum FT_4_ was significantly positively associated with aggression in this patient population (*β* = 0.244, 95% CI [0.148, 0.334], *p* < 0.001). After hostile attribution bias and psychological resilience were incorporated into the structural equation modeling, serum FT_4_ was significantly positively associated with hostile attribution bias (*β* = 0.272, 95% CI [0.182, 0.351], *p* < 0.001), and negatively associated with psychological resilience (*β* = −0.235, 95% CI [−0.324, −0.133], *p* < 0.001). In addition, hostile attribution bias was significantly negatively associated with psychological resilience (*β* = −0.370, 95% CI [−0.454, −0.279], *p* < 0.001). Hostile attribution bias was significantly positively associated with aggression among patients with thyroid disease (*β* = 0.473, 95% CI [0.378, 0.563], *p* < 0.001), whereas psychological resilience was significantly negatively associated with aggression (*β* = −0.288, 95% CI [−0.379, −0.197], *p* < 0.001).

On the basis of preliminary effect tests and path analysis, mediation effects were assessed using the bias-corrected nonparametric percentile bootstrapping procedure. The results, presented in Table 4, indicated the following mediation effects through four specific paths: the direct pathway (serum FT_4_—aggression), effect size 0.244, accounting for 51.91% of the total effect; the pathway via hostile attribution bias (serum FT_4_—hostile attribution bias—aggression), effect size 0.129, accounting for 27.45% of the total effect; the pathway via psychological resilience (serum FT_4_—psychological resilience—aggression), effect size 0.068, accounting for 14.47% of the total effect; and the serial pathway (serum FT_4_—hostile attribution bias—psychological resilience—aggression), effect size 0.029, accounting for 6.17% of the total effect. These results were consistent with the hypothesis that (1) serum FT_4_ levels are directly associated with aggression among thyroid disease patients, (2) hostile attribution bias plays a mediating role in the association between serum FT_4_ levels and aggression, (3) psychological resilience plays a mediating role in the association between serum FT_4_ levels and aggression, (4) hostile attribution bias and psychological resilience play a serial mediating role in the association between serum FT_4_ levels and aggression. Specifically, serum FT_4_ exhibited indirect associations with aggression through two independent mediating pathways (hostile attribution bias and psychological resilience), as well as the chain mediating pathway involving hostile attribution bias and psychological resilience.

**Table 4:** Mediating effect test of the relationship between serum FT_4_ and aggression.

Effect type	Pathway	*B*	*β*	95% CI		Proportion of total effect (%)
				Lower	Upper	
Direct effect	X → Y	0.160	0.244	0.148	0.334	51.91
Indirect effect	X → M1 → Y	0.084	0.129	0.088	0.178	27.45
	X → M2 → Y	0.044	0.068	0.038	0.107	14.47
	X → M1 → M2 → Y	0.019	0.029	0.016	0.048	6.17

X, serum FT_4_; M1, hostile attribution bias; M2, psychological resilience; Y, aggression; *B*, unstandardized coefficient; β, standardized coefficient; CI, confidence interval.

#### 3.3.2 Alternative model analyses

As shown in Table 3, all three models yielded identical overall fit indices. However, the internal pathways differed substantially (Supplementary Tables S3 and S4). In Alternative Model 1, the serial indirect pathway (serum FT_4_—psychological resilience—hostile attribution bias—aggression) was non-significant (95% CI [−0.0002, 0.0004]). In Alternative Model 2, the path from psychological resilience to hostile attribution bias (*β* = −0.077, *p* = 0.132), the path from hostile attribution bias to serum FT_4_ (*β* = −0.081, *p* = 0.198), the indirect pathway (aggression—psychological resilience—serum FT_4_; *β* = −0.050, *p* = 0.185) and the serial indirect pathway (aggression—psychological resilience—hostile attribution bias—serum FT_4_; *β* = −0.004, *p* = 0.143) were all non-significant. Although the three models were statistically equivalent in overall fit, only the initial hypothesized model yielded a significant serial mediation pathway (serum FT_4_—hostile attribution bias—psychological resilience—aggression; *β* = 0.029, 95% CI [0.016, 0.048]).

#### 3.3.3 Sensitivity analyses

As shown in Supplementary Table S5, all adjusted models demonstrated acceptable fit. The significance and magnitude of direct and indirect effects remained largely unchanged after adjusting for TSH, T_3_, and FT_3_ (Supplementary Tables S6 and S7). The serial indirect pathway was *β* = 0.029 (95% CI [0.016, 0.048]) in the initial model, compared with *β* = 0.028 (95% CI [0.016, 0.047]), *β* = 0.029 (95% CI [0.016, 0.047]), and *β* = 0.030 (95% CI [0.017, 0.051]) in the respective TSH-, T_3_-, and FT_3_-adjusted models. The 95% confidence intervals for all indirect pathways across all adjusted models consistently excluded zero. Overall, these findings suggested that the hypothesized serial mediation model was not materially confounded by TSH, T_3_, or FT_3_.

#### 3.3.4 Sex-stratified multi-group analysis

As shown in Supplementary Table S8, constraining measurement weights (ΔCFI = 0.001, ΔRMSEA = −0.004) and structural weights (ΔCFI = −0.003, ΔRMSEA = −0.010) yielded negligible fit changes, meeting invariance criteria (|ΔCFI| ≤ 0.01, ΔRMSEA ≤ 0.015). The Chi-square difference test between the unconstrained and structural weights models was non-significant (*p* = 0.068; Supplementary Table S9). Additionally, sex-stratified path coefficients (Supplementary Table S10) showed overlapping 95% CIs for all pathways. The serial indirect pathway via hostile attribution bias and psychological resilience was *β* = 0.035 (95% CI [0.007, 0.039]) for males and *β* = 0.017 (95% CI [0.003, 0.036]) for females. Based on the sex-stratified sample (44% male, 56% female; *α* = 0.05), post-hoc power analysis (Supplementary Table S11) indicated power values of 0.999 for medium effects, 1.000 for large effects, and 0.525 for small effects (Cohen’s *d* = 0.2). Overall, the model demonstrated cross-sex comparability without statistically meaningful moderation by sex, although the inadequate power for small effects precludes ruling out minimal between-sex differences.

## 4 Discussion

This study examined the association of serum FT_4_ with aggression, as well as the mediating roles of hostile attribution bias and psychological resilience. Before interpreting the empirical findings, it is important to clarify the interpretative boundaries of this cross-sectional analysis. All estimated pathways reflect statistical covariation rather than unidirectional causal effects, and definitive causal conclusions cannot be drawn from single-timepoint correlational data. The serial mediation model—which positions hostile attribution bias as the earlier mediator and psychological resilience as the subsequent mediator—was constructed *a priori* based on the SIP model (Crick and Dodge, 1994) and the GAM (Allen et al., 2018). To evaluate the rationality of this theoretically driven sequential ordering, two alternative competing models were constructed for comparison: the first reversed the order of the two mediators, and the second fully reversed the overall directional flow of the entire model. Critically, both alternative models yielded non-significant serial indirect effects. Although cross-sectional SEM inevitably generates statistically equivalent models with identical global fit indices (MacCallum et al., 1993) and reverse directional sequences remain theoretically conceivable, the present empirical evidence strongly corroborates the initial hypothesized chain as the only coherent and statistically supported ordering. This finding offers associational insights for unpacking the internal linkage between FT_4_ levels and aggressive behaviors in thyroid patients. Accordingly, the following discussion focused exclusively on interpreting this theoretically grounded, empirically supported framework.

To begin with, this study confirmed the research hypothesis H1: serum FT_4_ was significantly positively associated with aggression in thyroid disease patients. The findings showed a standardized direct effect of *β* = 0.244 (*p* < 0.001), and an unstandardized coefficient of *B* = 0.160, indicating that patients with higher FT_4_ levels had correspondingly higher BPAQ scores. The direct pathway accounted for 51.91% of the total effect, suggesting that the direct physiological association was the largest component of the FT_4_—aggression association in the hypothesized model. Although previous studies have explored the direct association between serum FT_4_ levels and human aggression—reporting higher FT_4_ levels in the control group (i.e., non-offenders, presumably with lower aggression) than in offenders (Stalenheim, 2004), identifying thyroid dysfunction as a correlate of aggression in major depressive disorder (Zhao et al., 2025), finding serum FT_4_ levels to be a risk factor for physical violence (Li et al., 2023), and suggesting that high FT_4_ levels may be associated with aggression-related crimes (Acar and Ulgen, 2020)—the present study was the first to report a positive association between FT_4_ levels and aggression specifically in thyroid disease patients.

Based on previous research, potential explanations for this association were discussed within the context of existing theories. The optimal serum FT_4_ levels are critical for mood regulation and have been found to bind to thyroid hormone receptors in the limbic system, and these interactions have been associated with antidepressant-related changes (Pedersen et al., 2007). Additionally, FT_4_ levels have been found to correlate with anxiety and depression, potentially in conjunction with their modulation of hippocampal brain-derived neurotrophic factor levels and brain-wide serotonergic signaling (Flach et al., 2021). Moreover, the association between depression, anxiety, and aggression has been documented in previous studies (Zhang et al., 2023; Chen et al., 2025). That aligns with the Mendelian randomization study by Kuś et al. (2021), which observes a nominally significant inverse association between FT_4_ levels and bipolar disorder risk. Significantly, the amygdala, which is associated with emotion generation, regulation, and impulse control, has been linked to serum FT_4_ levels. In summary, higher serum FT_4_ levels are associated with neural excitability in thyroid disease patients and may be correlated with disruptions in brain systems; such disruptions might co-occur with emotional dysregulation, including anger and depression, and these factors together show a positive association with aggression. These findings were consistent with the pattern that higher serum FT_4_ levels were associated with higher aggression in thyroid disease patients. However, the association between serum FT_4_ and aggression in these patients may be complicated, and more potential factors remain to be investigated. While this individual unit effect was modest in this study, thyroid disease patients frequently experience wide FT_4_ fluctuations during hyperthyroid episodes and drug titration. Long-term sustained FT_4_ elevation may be considered a potential correlate of impulsive aggression, an issue rarely addressed in routine endocrine follow-up. These findings raised the possibility that regular psychological screening for aggression and hostile symptoms may be relevant for patients with persistently elevated FT_4_. In addition, it appears worth considering combined endocrinological and psychological intervention strategies, given their possible statistical association with fewer aggressive adverse events.

From a cognitive research perspective, this study found that hostile attribution bias was positively associated with aggression and was identified as a significant statistical mediator between serum FT_4_ and aggression in thyroid disease patients, supporting hypothesis H2. Notably, the total score of the WSAP-Hostility scale in the present study was calculated as the total score of the hostile attribution subscale minus that of the benevolent attribution subscale. The mean hostile attribution bias score was −7.18, indicating that patients showed an overall higher level of benevolent attribution relative to hostile attribution. Several reasons may account for this finding. First, the participants enrolled in this study were inpatients diagnosed with thyroid diseases, rather than individuals with psychiatric disorders or impulse-control disorders frequently recruited in previous studies on hostile attribution bias. Second, the relatively large standard deviation (SD = 19.78) revealed prominent individual heterogeneity within the sample; a subset of participants still exhibited a high level of hostile attribution tendency, which was consistent with the positive predictive association between hostile attribution bias and aggressive behaviors even when the overall sample leaned toward benevolent social interpretation. In addition, the Cronbach’s *α* coefficients of the WSAP-Hostility scale in this study were 0.743 for the total scale, 0.806 for the hostile attribution subscale, and 0.793 for the benevolent attribution subscale, which demonstrated satisfactory internal consistency. This finding verified the stable and reliable psychometric properties of this instrument among thyroid-diseased inpatients, enabling it to effectively distinguish individual differences in attribution tendencies and objectively capture the association between hostile attribution bias and aggression. The indirect pathway via hostile attribution bias accounted for 27.45% of the total effect of FT_4_ on aggression, ranking first among all indirect mediating pathways. This result suggested that hostile attribution bias was the most prominent theoretical mediating pathway that statistically accounted for a substantial portion of the FT_4_–aggression association in this model. Clinically, correcting hostile cognitive bias may be a relevant intervention target in efforts to address aggressive risk among thyroid disease patients. Collectively, these results were in line with the view that thyroid disease patients with higher serum FT_4_ levels tended to show higher hostile attribution bias, which in the hypothesized sequence corresponded to higher aggression. Nevertheless, normative data for patients with thyroid diseases are currently unavailable, which indicates that future research should develop hostile attribution bias scales specifically calibrated for patients with endocrine disorders, and these normative data need to be further supplemented and validated in future research.

This finding aligns with the randomized controlled trial study by Liu et al. (2024), which discovered that hostile attribution bias is positively associated with human aggression. Also, an explicitly positive correlation between hostile attribution bias and aggression has been indicated in previous research (Bondü and Richter, 2016; Gagnon and Rochat, 2017). In addition, this result was supported by the SIP Model (Crick and Dodge, 1994), which describes how hostile attribution bias is theoretically linked to reactive aggression. The SIP model outlines that if an individual misinterprets others’ behaviors as hostile, he or she may select aggressive behaviors as appropriate responses to perceived provocation (Dill et al., 1997). First, serum FT_4_ levels are associated with amygdala reactivity to threatening stimuli and this reactivity has been associated with aggression. Given the amygdala’s important role in the generation and regulation of emotions, its dysfunction has been associated with hostile attribution bias (Yang and Wang, 2017). Secondly, FT_4_ levels have been found to show a positive association with executive function, which in turn is strongly correlated with hostile attribution bias (van Vliet et al., 2021). While previous research has not fully established a link between serum FT_4_ and hostile attribution bias, this study observed such a connection and further identified the mediating role of hostile attribution bias in the association between serum FT_4_ and aggression in thyroid disease patients, offering a basis for future theoretical refinements. Notably, the unique characteristics of the hospital setting may influence the expression of hostile attribution bias.

From a psychological research perspective, this study revealed that psychological resilience was negatively associated with aggression and significantly mediated the relationship between serum FT_4_ and aggression in thyroid disease patients, thus supporting hypothesis H3. The indirect pathway through psychological resilience accounted for 14.47% of the total effect. Although statistically significant, this pathway had weaker explanatory power than hostile attribution bias, indicating psychological resilience as a secondary protective factor. Clinically, boosting psychological resilience can serve as an auxiliary intervention to reduce aggression in thyroid disease patients. The observations were in line with the conclusions reported in previous relevant studies (Acar and Ulgen, 2020) which finds that psychological stress may affect the hypothalamus-pituitary-thyroid axis, and that thyroid hormone levels may be related to aggression. Firstly, as an internal protective factor, psychological resilience plays a positive role in resisting stress from diseases and enhancing adaptability and recovery. To a certain extent, it has a positive buffering effect against the negative impacts associated with the increase in serum FT_4_ levels (Troy et al., 2023). Secondly, according to the GAM (Allen et al., 2018), psychological resilience functions not merely as an emotional state associated with aggression but as a critical internal process that is involved in shaping behavioral responses. Furthermore, with various biological correlates, such as alterations in neurotransmitters and exhaustion of the noradrenergic system (Bunevicius and Prange, 2010), high FT_4_ levels are associated with an increased risk of depression in thyroid disease patients (Delitala et al., 2016), which in turn may be related to reduced psychological resilience and heightened aggression. This was consistent with previous cross-sectional and longitudinal studies, which have demonstrated an association between high FT_4_ levels and more depressive symptoms (Roa Dueñas et al., 2024).

In addition, this study found that hostile attribution bias was negatively associated with psychological resilience, and that the two factors serially mediated the relationship between serum FT_4_ and aggression in thyroid disease patients, thereby supporting hypothesis H4. The serial indirect pathway only accounted for 6.17% of the total effect, the weakest among all indirect pathways, suggesting that this cognitive-emotional chain accounted for a limited portion of the statistical association between FT_4_ and aggression. Correlations among the variables indicated that the serum FT_4_ levels were significantly and positively associated with hostile attribution bias, and that the hostile attribution bias was negatively correlated with psychological resilience. This finding aligns with previous research suggesting that adolescents with lower levels of hostility bias exhibit higher levels of psychological resilience (Iselin et al., 2025). According to the GAM (Allen et al., 2018), individuals under negative stress are influenced by input variables (individual factors and situational factors) and pathways (cognitive processes and emotional processes), ultimately manifesting as output variables, including violence and escape. This model provides a useful framework for understanding this chain mediating effect: the increase in serum FT_4_ levels serves as the input variable, with hostile attribution bias and psychological resilience acting as the cognitive and emotional pathways, respectively. Together, they were statistically correlated with aggression, which was the final outcome within the model. Specifically, serum FT_4_ levels are specified to interact with thyroid hormone receptors in the amygdala and are associated with brain-wide neurotransmitter transmission, which may in turn relate to hostile attribution bias (Ritchie and Yeap, 2015). Besides, individuals with high hostile attribution bias are prone to misinterpreting objective information, falling into negative emotions, and engaging in negative rumination, which may ultimately be associated with decreased psychological resilience and increased likelihood of engaging in aggression (Quan et al., 2022; Subra, 2023). Conversely, patients with low levels of hostile attribution bias tend to perceive more social support in interactions, which corresponds to higher psychological adaptation, fewer negative emotions, and lower aggression.

In summary, this study took thyroid disease patients as an example to investigate the association of serum FT_4_ with human aggression and analyzed the chain mediating roles of hostile attribution bias and psychological resilience in this association. The results further provided insights into the potential pathways underlying the association between serum FT_4_ levels and aggression and provided a more comprehensive scientific and empirical basis for designing intervention programs for aggression in thyroid disease patients.

## 5 Limitations

The results obtained in this study were promising in terms of theory and practice, although it was necessary to point out certain limitations. Firstly, and most importantly, although the mediation model was theory-driven, the cross-sectional design precludes causal inferences and the proposed pathways should therefore be interpreted as a theoretically consistent pattern of associations rather than evidence of causal sequencing (Kesmodel, 2018). Future longitudinal or experimental studies are needed to establish temporal precedence. Secondly, social desirability is considered one of the most influential biases in responses when a cross-sectional design involves self-report questionnaires (Holtgraves, 2004). Thirdly, we recruited inpatients from a single hospital, and certain clinical variables were not fully collected, which may moderately restrict confounding adjustment and the interactive relationship between variables. Additionally, hospitalization stress itself may introduce physiological and psychological changes that could affect both thyroid function and aggression, and this factor may act as a potential confounder. Furthermore, although the cross-sectional design does not require a control group for internal validity, the absence of a healthy control prevents direct comparison of effect magnitudes between thyroid diseases and non-thyroid populations. Future multi-center research with broader indicators and matched control groups could validate the conclusions. In addition, there may be other mediating variables other than those assessed in this study that have not been included in this research hypothesis and require further exploration in the future.

## 6 Conclusion

Overall, the results of this study not only demonstrated the unique associations of the thyroid hormones with human aggression but also broadened the understanding of potential pathways in the association between serum FT_4_ and aggression in thyroid disease patients. As cognitive and psychological factors, hostile attribution bias and psychological resilience, respectively, served as independent and serial mediators in the association between serum FT_4_ and aggression. Sex-stratified multi-group structural equation modeling revealed that no significant sex differences were observed in the pathways from FT_4_ to aggression.

Moreover, the results of this study contribute to the development of interventional programs aimed at reducing patients’ aggression during medical treatment in future studies. On the one hand, monitoring serum FT_4_ in patients may help identify individuals at higher risk for aggressive behavior. On the other hand, the joint assessment of hostile attribution bias and psychological resilience might facilitate the early identification of patients’ aggression.

### 6.1 Relevance for clinical practice

Given that this was a cross-sectional study, the following interpretations were based on associations rather than causal effects. Based on the findings of this study, greater attention should be paid to aggression among patients with thyroid diseases in clinical practice. Lower serum FT_4_ levels, reduced hostile attribution bias and enhanced psychological resilience were each correlated with lower aggression in our sample. Clinically, improved communication skills to ensure accurate and effective information exchange may correlate with lower hostile cognitive biases of patients. In addition, psychological counseling and supportive interventions may be associated with higher psychological resilience and lower aggression levels. For patients with elevated levels of FT_4_, providing strategies for managing cognitive and psychological functioning may correlate with less biased social cognition and better resilience, which were both correlated with weaker aggressive tendencies in our sample. The findings of this study may provide a theoretical reference for further research on factors associated with aggression among thyroid disease patients and on factors relevant to harmonious relationships between medical staff and patients.

## Data Availability

The original contributions presented in the study are included in the article/Supplementary material, further inquiries can be directed to the corresponding authors.
