# Supplementary material for: Association between free thyroxine and aggression: the chain mediating role of hostile attribution bias and psychological resilience
Source: Front Psychol. 2026 Jul 30;17:1822609. doi: 10.3389/fpsyg.2026.1822609 (PMC13469276; doi:10.3389/fpsyg.2026.1822609)
Supplement: Supplementary file 1 [file Supplementary_file_1.DOCX]

TABLE S1 Comparison of study variables across groups (N=418).

| Variables | Category | Number of patients (%) | BPAQ scores | *F* | *P* |
| --- | --- | --- | --- | --- | --- |
|  |  |  | M ± SD |  |  |
| FT_4_ (pmol/L) | Below normal | 55(13.16) | 60.75 ± 16.43 | 17.833 | < 0.001 |
|  | Normal | 328(78.47) | 68.63 ± 16.35 |  |  |
|  | Above normal | 35(8.37) | 82.94 ± 24.93 |  |  |
| TSH(mIU/L) | Below normal | 28(6.70) | 72.07 ± 18.20 | 2.246 | 0.107 |
|  | Normal | 371(88.75) | 68.94 ± 19.95 |  |  |
|  | Above normal | 19(4.55) | 61.11 ± 15.16 |  |  |
| T_3_(ng/mL) | Below normal | 106(25.36) | 67.28 ± 14.28 | 1.498 | 0.225 |
|  | Normal | 304(72.73) | 69.53 ± 19.04 |  |  |
|  | Above normal | 8(1.91) | 60.50 ± 14.18 |  |  |
| FT_3_(pmol/L) | Below normal | 95(22.73) | 69.96 ± 15.46 | 1.767 | 0.172 |
|  | Normal | 318(76.08) | 68.23 ± 18.26 |  |  |
|  | Above normal | 5(1.19) | 82.20 ± 33.09 |  |  |
| HAB | Low | 101(24.16) | 56.22 ± 13.27 | 57.514 | < 0.001 |
|  | Medium | 310(74.16) | 72.04 ± 16.65 |  |  |
|  | High | 7(1.68) | 106.14 ± 15.13 |  |  |
| PR | Low | 38(9.09) | 93.45 ± 24.85 | 64.494 | < 0.001 |
|  | Medium | 313(74.88) | 68.20 ± 15.09 |  |  |
|  | High | 67(16.03) | 57.54 ± 11.05 |  |  |

FT_4_, free thyroxine; TSH, thyroid-stimulating hormone; T_3_, triiodothyronine; FT_3_, free triiodothyronine; HAB, hostile attribution bias; PR, psychological resilience; M, mean; SD, standard deviation.

TABLE S2 Sensitivity analysis for trust–aggression comparisons excluding the “Completely Distrustful” group (n = 5).

| Trust Category | Original Analysis | | Sensitivity Analysis | |
| --- | --- | --- | --- | --- |
|  | Number of patients (%) | BPAQ scores | Number of patients (%) | BPAQ scores |
|  |  | M ± SD |  | M ± SD |
| Completely Distrustful | 5 (1.20) | 115.60 ± 3.36 | – | – |
| Somewhat Distrustful | 19 (4.55) | 100.79 ± 11.56 | 19 (4.60) | 100.79 ± 11.56 |
| Neutral | 39 (9.33) | 86.77 ± 13.99 | 39 (9.44) | 86.77 ± 13.99 |
| Somewhat Trustful | 160 (38.27) | 70.39 ± 14.57 | 160 (38.74) | 70.39 ± 14.57 |
| Completely Trustful | 195 (46.65) | 59.56 ± 12.43 | 195 (47.22) | 59.56 ± 12.43 |
| Overall | 418 | 68.79 ± 17.90 | 413 | 68.22 ± 17.25 |
| F (*p*) |  | 84.21(< 0.001) |  | 90.72(< 0.001) |

TABLE S3 Mediating effect test of the relationship between serum FT_4_ and aggression in Alternative Model 1.

| Pathway | *β* | 95% CI | | *P* |
| --- | --- | --- | --- | --- |
|  |  | Lower | Upper |  |
| Serum FT_4_→Aggression | 0.244 | 0.148 | 0.334 | < 0.001 |
| Serum FT_4_→Psychological resilience | -0.336 | -0.428 | -0.224 | 0.002 |
| Serum FT_4_→Hostile attribution bias | 0.143 | 0.065 | 0.218 | < 0.001 |
| Psychological resilience→Hostile attribution bias | -0.386 | -0.470 | -0.292 | < 0.001 |
| Psychological resilience→Aggression | -0.288 | -0.380 | -0.197 | < 0.001 |
| Hostile attribution bias→Aggression | 0.473 | 0.377 | 0.561 | < 0.001 |
| Serum FT_4_→Psychological resilience→Aggression | 0.067 | 0.031 | 0.111 | 0.002 |
| Serum FT_4_→Hostile attribution bias→Aggression | 0.097 | 0.060 | 0.146 | < 0.001 |
| Serum FT_4_→Psychological resilience→Hostile attribution bias→Aggression | 0.0001 | -0.0002 | 0.0004 | 0.514 |

FT_4_, free thyroxine; *β,* standardized coefficient; CI, confidence interval.

TABLE S4 Mediating effect test of the relationship between serum FT_4_ and aggression in Alternative Model 2.

| Pathway | *β* | 95% CI | | *P* |
| --- | --- | --- | --- | --- |
|  |  | Lower | Upper |  |
| Aggression→Serum FT_4_ | 0.464 | 0.282 | 0.629 | < 0.001 |
| Aggression→Psychological resilience | -0.575 | -0.657 | -0.485 | < 0.001 |
| Aggression→Hostile attribution bias | 0.620 | 0.513 | 0.719 | < 0.001 |
| Psychological resilience→Hostile attribution bias | -0.077 | -0.176 | 0.023 | 0.132 |
| Psychological resilience→Serum FT_4_ | -0.104 | -0.197 | -0.005 | < 0.001 |
| Hostile attribution bias→Serum FT_4_ | -0.081 | -0.207 | 0.044 | 0.198 |
| Aggression→Psychological resilience→Serum FT_4_ | -0.050 | -0.139 | 0.025 | 0.185 |
| Aggression→Hostile attribution bias→Serum FT_4_ | 0.060 | 0.005 | 0.115 | 0.032 |
| Aggression→Psychological resilience→Hostile attribution bias→Serum FT_4_ | -0.004 | -0.016 | 0.001 | 0.143 |

FT_4_, free thyroxine; *β,* standardized coefficient; CI, confidence interval.

TABLE S5 Comparisons of model fit indices before and after controlling for TSH, T_3_, and FT_3_.

| Index | *χ^2^/df* | RMSEA | NFI | IFI | TLI | CFI |
| --- | --- | --- | --- | --- | --- | --- |
| Evaluation Indicators | < 3.00 | < 0.08 | > 0.90 | > 0.90 | > 0.90 | > 0.90 |
| Initial Model | 2.827 | 0.047 | 0.951 | 0.968 | 0.937 | 0.967 |
| TSH-adjusted Model | 2.973 | 0.069 | 0.956 | 0.971 | 0.940 | 0.970 |
| T_3_-adjusted Model | 3.164 | 0.072 | 0.953 | 0.968 | 0.934 | 0.967 |
| FT_3_-adjusted Model | 3.184 | 0.072 | 0.955 | 0.969 | 0.937 | 0.968 |

*χ*^2^, Chi-square; *df*, Degrees of freedom; RMSEA, Root Mean-Square Error of Approximation; NFI, Normed Fit Index; IFI, Incremental Fit Index; TLI, Tucker-Lewis Index; CFI, Comparative Fit Index; TSH, thyroid-stimulating hormone; T_3_, triiodothyronine; FT_3_, free triiodothyronine.

TABLE S6 Comparisons of standardized path coefficients before and after controlling for TSH, T_3_, and FT_3_.

| Pathway | Initial Model | | | TSH-adjusted Model | | | | T_3_-adjusted Model | | | | FT_3_-adjusted Model | | | |
| --- | --- | --- | --- | --- | --- | --- | --- | --- | --- | --- | --- | --- | --- | --- | --- |
|  | *β* | 95% CI | | *β* | 95% CI | | \|Δ*β\|* | *β* | 95% CI | | \|Δ*β\|* | *β* | 95% CI | | \|Δ*β\|* |
|  |  | Lower | Upper |  | Lower | Upper |  |  | Lower | Upper |  |  | Lower | Upper |  |
| X→Y | 0.244 | 0.148 | 0.334 | 0.241 | 0.147 | 0.330 | 0.003 | 0.252 | 0.156 | 0.341 | 0.008 | 0.257 | 0.156 | 0.353 | 0.013 |
| X→M1 | 0.272 | 0.182 | 0.351 | 0.273 | 0.183 | 0.352 | 0.001 | 0.270 | 0.180 | 0.351 | 0.002 | 0.287 | 0.190 | 0.372 | 0.015 |
| X→M2 | -0.235 | -0.324 | -0.133 | -0.233 | -0.321 | -0.132 | 0.002 | -0.239 | -0.328 | -0.136 | 0.004 | -0.257 | -0.354 | -0.148 | 0.022 |
| M1→M2 | -0.370 | -0.454 | -0.279 | -0.370 | -0.454 | -0.278 | 0 | -0.371 | -0.452 | -0.277 | 0.001 | -0.368 | -0.450 | -0.274 | 0.002 |
| M1→Y | 0.473 | 0.378 | 0.563 | 0.476 | 0.382 | 0.565 | 0.003 | 0.476 | 0.380 | 0.563 | 0.003 | 0.473 | 0.377 | 0.561 | 0 |
| M2→Y | -0.288 | -0.379 | -0.197 | -0.279 | -0.370 | -0.186 | 0.001 | -0.285 | -0.375 | -0.192 | 0.003 | -0.286 | -0.380 | -0.194 | 0.002 |

X, serum free thyroxine; M1, hostile attribution bias; M2, psychological resilience; Y, aggression; TSH, thyroid-stimulating hormone; T_3_, triiodothyronine; FT_3_, free triiodothyronine; *β,* standardized coefficient; CI, confidence interval.

TABLE S7 Comparisons of standardized mediation effects before and after controlling for TSH, T_3_, and FT_3_.

| Pathway | Initial model | | | TSH-adjusted Model | | | | T_3_-adjusted Model | | | | FT_3_-adjusted Model | | | |
| --- | --- | --- | --- | --- | --- | --- | --- | --- | --- | --- | --- | --- | --- | --- | --- |
|  | *β* | 95% CI | | *β* | 95% CI | | \|Δ*β\|* | *β* | 95% CI | | \|Δ*β\|* | *β* | 95% CI | | \|Δ*β\|* |
|  |  | Lower | Upper |  | Lower | Upper |  |  | Lower | Upper |  |  | Lower | Upper |  |
| X→M1→Y | 0.129 | 0.088 | 0.178 | 0.130 | 0.088 | 0.181 | 0.001 | 0.129 | 0.087 | 0.179 | 0 | 0.136 | 0.092 | 0.191 | 0.007 |
| X→ M2 →Y | 0.068 | 0.038 | 0.107 | 0.065 | 0.036 | 0.103 | 0.003 | 0.068 | 0.038 | 0.107 | 0 | 0.073 | 0.041 | 0.118 | 0.005 |
| X→M1→M2→Y | 0.029 | 0.016 | 0.048 | 0.028 | 0.016 | 0.047 | 0.001 | 0.029 | 0.016 | 0.047 | 0 | 0.030 | 0.017 | 0.051 | 0.001 |

X, serum free thyroxine; M1, hostile attribution bias; M2, psychological resilience; Y, aggression; TSH, thyroid-stimulating hormone; T_3_, triiodothyronine; FT_3_, free triiodothyronine; *β,* standardized coefficient; CI, confidence interval.

TABLE S8 Multi-group tests of measurement and structural invariance across sexes.

| Model | *χ*² | *df* | CFI | RMSEA | ΔCFI | ΔRMSEA |
| --- | --- | --- | --- | --- | --- | --- |
| Unconstrained | 93.291 | 33 | 0.967 | 0.047 | — | — |
| Measurement weights | 97.756 | 39 | 0.968 | 0.043 | 0.001 | -0.004 |
| Structural weights | 124.952 | 59 | 0.964 | 0.037 | -0.003 | -0.010 |

*χ*^2^, Chi-square; *df*, Degrees of freedom; CFI, Comparative Fit Index; RMSEA, Root Mean-Square Error of Approximation.

Note: ΔCFI and ΔRMSEA represent changes relative to the unconstrained model. Criteria for invariance: |ΔCFI| ≤ 0.01 and ΔRMSEA ≤ 0.015.

TABLE S9 Nested invariance test across sexes.

| Model | DF | CMIN | *P* | NFI | IFI | RFI | TLI |
| --- | --- | --- | --- | --- | --- | --- | --- |
|  |  |  |  | Delta-1 | Delta-2 | rho-1 | rho2 |
| Constrained | 9 | 15.924 | 0.068 | 0.017 | 0.017 | -0.008 | -0.008 |

DF, Degrees of freedom; CMIN, Chi-square; NFI, Normed Fit Index; IFI, Incremental Fit Index; RFI, Relative Fit Index; TLI, Tucker-Lewis Index.

TABLE S10 Sex-stratified multi-group analysis.

| Pathway | Males | | | | Females | | | |
| --- | --- | --- | --- | --- | --- | --- | --- | --- |
|  | B | *β* | 95% CI | | B | *β* | 95% CI | |
|  |  |  | Lower | Upper |  |  | Lower | Upper |
| X → Y | 0.150 | 0.268 | 0.131 | 0.404 | 0.123 | 0.159 | 0.026 | 0.291 |
| X → M1 → Y | 0.096 | 0.172 | 0.051 | 0.187 | 0.062 | 0.081 | 0.017 | 0.131 |
| X → M2 → Y | 0.029 | 0.051 | 0.011 | 0.056 | 0.049 | 0.063 | 0.012 | 0.102 |
| X → M1 → M2→ Y | 0.019 | 0.035 | 0.007 | 0.039 | 0.013 | 0.017 | 0.003 | 0.036 |

X, serum free thyroxine; M1, hostile attribution bias; M2, psychological resilience; Y, aggression; B, unstandardized coefficient; *β,* standardized coefficient; CI, confidence interval.

TABLE S11 Statistical post-hoc power estimation based on sex-stratified sample sizes (44% male and 56% female, α = 0.05).

| Effect Size (Cohen’s *d*) | Power |
| --- | --- |
| Small (0.2) | 0.525 |
| Medium (0.5) | 0.999 |
| Large (0.8) | 1.000 |

Note: Adequate statistical power is defined as ≥ 0.80.
